# Infant Feeding*Read before the Atlanta Society of Medicine.

**Published:** 1898-08

**Authors:** G. P. Robinson

**Affiliations:** Atlanta, Ga.


### INFANT FEEDING.*

By G. P. ROBINSON, M.D.,

ATLANTA, GA.

To prevent disease is the highest province of every practitioner. The study of the best means for starting young life is the highest branch of preventive medicine.

With this objective point in view, the preventive medicine of young life consists in the proper and intelligent management of the nutriment which enables it to grow and to live. Thus the feeding and the knowledge of the proper preparation of such food becomes of paramount importance.

The subject of infant feeding is a broad one and should be treated broadly. It has many varied aspects; what agrees with one child is almost poison to another. To-day, through the study of many prominent investigators, our knowledge of infant feeding has been much increased and brought much nearer to an exact science. Yet we still have much to learn. It is my hope this evening to bring before you a description of the principles of infant feeding and the possibilities of substitute feeding.

I fully realize the difficulties that are to be encountered in edu-

*Read before the Atlanta Society of Medicine.

cating the public to such radical changes. The old method of changing the food to this or that because the present food does not agree with the child must, however, disappear if the profession at large will only insist that such changes shall be made only on a scientific basis. The great variety of artificial foods on the market and used by the physicians of to-day only goes to show that artificial feeding has not arrived at the point where it can compete with breast feeding. Many infants thrive on peculiar mixtures, and again many infants will not thrive on the food that nature has given them. The young infant is essentially a carnivora for its first twelve months of life, and therefore it is evident that an animal food freshly derived from animal, and not vegetable, sources is the best for the young lives.

*The Mammary Glands.*—You will pardon me if I revive your memory in regard to the mammary gland. I quote verbatim from Foster's Physiology: "The mammary gland is a compound racemose gland, and consists of a number of lobes composed of smaller divisions of lobules, which in turn are still subdivided in vesicles which form a cluster on one of the terminal ducts. They are composed of a basement membrane which is lined with glandular epithelium. Each lobule has a common duct, which, uniting with others, form some fifteen or twenty large ducts—the lactiferous ducts." So much for the partial anatomy of the gland. The milk is the result of the activity of certain protoplasmic secretic cells forming the epithelium of the mammary gland. As to the fat of milk, the processes taking place in the gland are very instructive. The fat can be seen to be gathered in the epithelium cell in the same way as a fat cell of the adipose tissue and to be discharged into the channels of the gland either by breaking away from the cell or by a contractile extension similar to that which takes place when an ameba ejects its digested food. All the evidence we possess goes to prove that the fat is found in the cell through a metabolism of the protoplasm. The microscopic history is supported by other facts, thus the quantity of fat present in milk is largely and directly increased by proteids, but not increased (on the contrary, diminshed) by fatty food. This is intelligible when we know that proteid food increases and fatty food diminishes the metabolism of the body. A bitch fed on meat for a given time gave off more fat in her milk than she could possibly have taken in her food, and that while she was gaining in weight, so that she could not have supplied the fat at the expense of fat previously existing in her body. She obtained it apparently from the proteids of her food. We have also indications that the casein is, like fat, formed in the cells of the gland, and not simply separated from the blood. When the action of the cell is imperfect, as at the beginning or end of lactation, the albumen in milk is in excess of the casein, but so long as the cell possesses its proper activity the formation of casein becomes prominent. That the milk-sugar is formed in and by the protoplasm of the cell is indicated by the fact that it is found in no other part of the body, and that its presence in milk is not dependent on carbohydrate food, for it is maintained in abundance in the milk of carnivora when they are fed exclusively on meat as free as possible from any kind of sugar or glycogen. We thus have evidence in the mammary gland of the formation by the direct metabolic activity of the secreting cell of the representatives of the three great classes of food-stuffs: proteids, fats, and carbohydrates, out of the comprehensive substance protoplasm. That both the secretion and ejection of milk are under the control of the nervous system is shown by common experience. These physiological facts become of interest and of the utmost importance when we attempt to modify or change the product of the gland.

A word now of the mammary gland in a general way. The breasts, when in an absolutely normal condition, are a most beautiful living machine, producing, measuring, and giving out a finished product capable of sustaining the life and continuing the growth of its young consumer. Again, the structure of this organ is of so delicate a character that any change of atmosphere, change of food, emotions, fatigue, catamenia, pregnancy, sickness may change the proportions of this finished product, and instead of its being a perfect food it becomes a very imperfect food.

It is not necessary for me to speak of the superiority of maternal feeding nor of the contraindications to maternal feeding, nor of the care of the mother and breasts, but I would like to say just a word in regard to the breast-pump.

There is a great difference of opinion in regard to the use of the breast-pump. Personally, I am strongly opposed to its use in ordinary cases when the breasts are to be emptied, but for its occasional use in securing samples of milk for analysis, I consider the end decidedly justifies the means.

Having considered the breast as a gland and its physiological functions, let us briefly look at its finished product, human milk and its clinical aspect. Unquestionably, in the great majority of cases, breast feeding is the *sine qua non.* Why? Because nature has so regulated the breasts that the quality and quantity is the best milk for each age and condition of each individual child. It must be clearly borne in mind that it is not breast milk as a whole that makes it the best, but the varied combination of the different elements of that milk. From the analysis of König, Foster, Meigs, Harrington and others, of the human milk of many women of different nationalities, the average analysis of human milk is found to be as follows:

| | |
|---|---|
| Reaction | Slightly alkaline. |
| Specific gravity | 1028–34, depending on temp. |
| Water | 87–88. |
| Total solids | 13–12. |
| Fat | 3–4 per cent. |
| Sugar | 6–7. |
| Proteids | 1–2. |
| Total ash | 0.1–0.2. |

This is simple and the constituents are few and easily grasped, and for our purpose this evening there is no necessity for going more minutely into their constituents.

Our knowledge of the human breast has shown us that the quantity for each succeeding age of the infant is regulated by the breast itself, so that if we find an infant nursing longer than fifteen or twenty minutes before the child is satisfied, it should make us suspicious that the milk is lacking in quantity; or, again, flabby breasts, or the desire of the infant for too frequent nursing, should make us look after the quantity.

Not so easy always is the determination of the quality of such milk. Clinical observation teaches that two things must be considered in the management of infant feeding—viz., the digestion of the infant and its nutrition. A milk may be easily digested but not nutritious, or again it may be nutritious and not easily digested, or it may be neither nutritious nor easily digested. It is the recognition and regulation of these conditions in the mother that the physician must be ready to meet. Much can be done toward this by the regulation of the diet and exercise, accurately and intelligently aided by repeated analyses of the milk at each step. Just here let me mention one of my cases that occurred to me in general practice illustrative of what changes can be made in breast milk. A Mrs. ——, multipara, whose infant was five months old. The child had thrived for the first two months of its life, but later began vomiting its food and was not increasing in weight. An analysis was made of the mother's milk (the exact per cent. I have not got), and the result showed a very low per cent. of fat and a very high per cent. of proteids. She was advised to eat meat and walk. Seen a week later, there was little or no improvement—there was plenty of milk, but no improvement in the child. On inquiry I found but little meat had been eaten—one small piece per diem—and not sufficient exercise had been taken. She was told to eat meat twice and walk at least three miles each day. From that on there was steady improvement, the child stopped vomiting, and there was steady increase in weight. An examination of the milk at this time showed, fat, 3.50; sugar, normal, and proteids reduced to 2 per cent., as nearly as I can remember.

Just to show the unusual percentages that a breast-fed child will at times thrive upon, let me quote a case of Dr. Rotch: A primipara; infant thriving, but as a matter of precaution in case of artificial feeding having to be resorted to, an analysis was made, with the following result:

| | |
|---|---|
| Fat | 5.16 |
| Sugar | 5.68 |
| Proteids | 4.14 |
| Ash | .17 |
| Total | 15.15 |
| Water | 84.85 |
| | 100.00 |

The child thrived and did well on these unusual percentages.

A long paper might be written on the care, regulation, idiosyncrasies and changes of percentages of breast feeding, and I have given it considerable space that we may more intelligently take up the subject of substitute feeding.

Before taking up the subject of substitute feeding let me say just one word in regard to mixed feeding. It not infrequently happens that the mother's milk, while agreeing with the infant in every way, fails in quantity. In these cases an exact knowledge of these percentages in the mother's milk enables us to substitute its exact equivalent. If, again, the breast milk, while agreeing with the child, does not nourish sufficiently, here again we can raise the percentages lacking in the mother's milk to the requisite amount in the substitute food.

*Indirect Substitute Feeding.*—We know the occasion frequently arises in civilized communities for supplying the young life with food other than breast milk. We have shown that children, for the first twelve months of life, are essentially carnivora, and that we are in no position to improve on nature's method of feeding. The food that approaches most nearly to the product of the human mamma is that produced from the mammal of other animals. Milk is the food that reason tells us is the proper substitute, and a milk that most nearly approaches to that of human milk. Without going into a discussion of the milks of different animals, it is a generally accepted fact that the product of the mamma of the cow is the most easily obtained, and though possibly not so closely resembling the constituents of human milk, yet, as all milk has to be modified, it is as easy to modify to a greater as to a less extent.

Accepting, then, the fact that cow's milk is the most available for substitute feeding, let us consider for a moment the breeds best adapted for our purpose. It is a well-known fact that the finer breeds from the Channel Islands are more liable to contract tuberculosis in our harsher climate than are the breeds represented by the Durham, Devon, or Holstein cattle. The Jerseys and Gurnseys, too, are somewhat richer in proteids, already too high in other breeds. It is also a fact that Jerseys cannot raise their own young. The old theory of one cow's milk is also fallacious, inasmuch as any disturbance of the equilibrium of the mammary glands through fright, bad feeding, or disease brings about a condition of the milk which the infant gets the full benefit of. The milk from a herd is the best.

The average analysis of cow's milk shows:

| | |
|---|---|
| Reaction | Slightly acid. |
| Specific gravity | 1029–33 |
| Water | 86-87 |
| Total solids | 14–13 |
| Fat | 4 per cent. |
| Sugar | 4.50 |
| Proteids | 4.00 |
| Ash | 70 |

This is an average analysis taken from a large number of common cows from all over the world, though it has been shown that by certain feeding of half ripened grasses a cow's milk may become neutral or alkaline, yet the ordinary-fed cattle produce an acid mixture.

Before speaking of the modification of cow's milk which must be made to make it correspond to human milk, let us briefly compare the two. The following table shows the percentages in woman's milk and cow's milk:

| | Woman's Milk. | Cow's Milk. |
|---|---|---|
| Reaction | Slightly alkaline. | As received, slightly acid. |
| Water | 87–88 | 86–87 |
| Total solids | 13-12 | 14–13 |
| Fat | 4 | 4 |
| Milk-sugar | 7 | 4.50 |
| Proteids | 1.50 | 4.00 |
| Ash | 0.20 | 0.7 |

*Reaction.*—As it is necessary to make both taste and reaction compare to mother's milk, and as the acidity of cow's milk is largely accountable for the large curds so frequently seen, it becomes essential that it be made slightly alkaline. Harrington has found by experiment that 6.25 per cent. of lime-water will produce an alkalinity corresponding to that of mother's milk.

*Water.*—There is one per cent. less water in cow's milk than in human milk. Such a large percentage of water in all human milk shows that nature intended a highly diluted food to be the best fitted for the young life.

*Fat.*—The percentages of fat of both the average human and cow's milk is the same, so that for modification we regulate only the fat as a whole.

*Sugar.*—The sugar of all mammals is of the kind known as milk-sugar or lactose. The percentages in the two kinds of milk, you will notice, are quite different, that of human milk being about 7 per cent. and that of cow's milk about 4.50. In modification this can be easily remedied by adding the milk-sugar.

*Proteids.*—The proteids in human milk have quite a wide range, yet, considering it between one and two, it can be stated that the relation of the percentages between cow's milk and human milk is as 4 to 1.5. The proteids represent the nitrogenous elements of milk. The coagulable proteids in cow's milk, being larger than in human milk, under the same conditions, a larger curd will be produced by the former than the latter. Harrington has worked out an extensive table showing the coagulability of milk by acetic acid. Woman's milk gives no curd perceptibly to the eye; cow's milk, raw, large curd. Cow's milk, 1 part; water, four parts, a fine curd. A modified mixture of fat, 4 parts; sugar, 7; proteids, 1.5; ash, 0.2; reaction, alkaline, gave a *very* fine curd, and water 5 parts, and milk 1 part, gave no curd perceptible to the eye. Cow's milk, direct from the udder, gave just as large a curd as that 24 hours old. Cow's milk, boiled, steamed or raw, also gave the same large curd; also, with lime-water and barley-water, the results were the same as with water. All this shows that the size of the curd depends on the dilution of the proteids rather than on any particular property of the substitute with which it is diluted. This is well to remember in substitute feeding—water is the best diluent.

Having seen how human milk is produced and how its percentages can be changed, and having accepted cow's milk as the best substitute, and having seen its constituents and percentages, I think you will agree with me that the mere technique of such modification must be simple. I also think that you will agree with me that the importance of modifying milk with the most exact precision is essential if we wish a perfect substitute food. Until, however, we can get a laboratory of the Walker-Gordon type (which I hope will not be long in coming here), the absolute perfection in the modifying of milk cannot be reached. In the "home modification," however, the percentages and constituents of cow's milk can be so changed and accurately carried out, if properly attended to, as to be all that is essential in the copying of the percentages of breast milk. The necessary articles for the modification of milk at home are simple and can be easily obtained here in Atlanta.

First, a simple sterilizer, containing a rack with some six or seven tubes; into the lid should be fitted a thermometer. The tubes are simple bottles, without curves so that they may be easily kept clean, and can be used as nursing-bottles. These are graduated and placed in the rack, and the rack in the sterilizer where the water comes to the top of the milk. Cotton wool for stoppers for these bottles.

A cozy—simply a woolen wrapper to retain the heat in the sterilizer after the lamp or stove has been taken from beneath.

A glass graduate of 8 ounces or more, divided into half-drachms.

Milk-sugar and lime-water.

A measure containing ʒ 3$\frac{3}{8}$. This measure does away with the necessity of the apothecary doing the sugar up into packages.

A siphon to separate the cream from the milk. This should be of glass and not rubber. To operate the siphon, fill it with boiled water, close the long end with the finger, place the short end in the milk, and, removing the finger, allow the water and then the milk to run out. Never use the lips to start the siphon. The mother should be told to follow your directions implicitly or a uniformly correct result will not be obtained. Cleanliness in regard to the cattle, and the obtaining the milk from a herd and not from one cow, as the percentages in the milk as a whole are less likely to vary, should be explained. The cows should be of a common breed, as we have seen. The milk should be thoroughly strained; after straining it should be placed in a quart jar, which is put in ice-cold water and allowed to stand, covered with a piece of freshly-boiled cotton cloth, for 15 or 20 minutes until the animal heat is exhausted. The jar is then sealed tightly as for preserving and allowed to stand for six hours in the ice water, care being used that the temperature of the water does not fall below 35 degrees, Fah. At the end of six hours siphon off your milk, and you now have your various materials ready for any combination you may wish to make. These are:

The milk you have siphoned off.

The cream, containing ten per cent. of fat, remaining in the jar.

The sugar.

Some fresh lime-water.

Some clean drinking-water which has been boiled five minutes.

After the various materials have been mixed in the proportions desired, the mixture is ready for the sterilizer. The requisite amount for each feeding is placed in the tubes and they are stopped with cotton wool, care being used to have it tight and the neck of the bottle dry. Then place the bottles in the rack and place it in the sterilizer, adjusting the water to the level of the milk in the tubes. Heat is then applied with the cover off until the water shows 171 degrees Fah. The cover is then replaced, the heat removed and the cozy wrapped about the sterilizer. The thermometer should then mark a temperature of between 167 and 170 degrees Fah. for thirty minutes. At the expiration of that time the tubes are to be removed and kept in a cool place until needed. On using the tubes they should be warmed to blood-heat and a clean nipple attached.

*Artificial Foods.*—One would think that the suggestion for the proper rules governing substitute feeding should emanate from the medical profession rather than from the non-medical proprietors of certain preparations. Yet, in looking over the great field of artificial feeding, the position occupied by the family physician, in comparison to the vendors of patent foods and proprietary artificial foods administered by nurses, is a most humiliating one, and one that should no longer exist. In view of the large amount of work done by our own profession, and especially by Holt and Rotch, to whom I am largely indebted for what I may know of infant feeding, we should certainly bury the statements of food proprietors who are constantly trying to stem the tide of progress.

The criticism has been made that modification is too expensive. Rotch, in this connection, suggests that human milk, which we are trying to copy, so far from being a cheap product, is a very expensive one.

The artificial foods are far from being reliable, and all of them, even when containing, as claimed in their circulars, certain percentages of fat, proteids, etc., when diluted for the infant's feeding, may have these constituents reduced below the limits of nutrition. Let us continue to march on until we have a perfect substitute and proprietary foods for substitute feeding become a thing of the past.

---

### THE RADICAL TREATMENT OF HYDROCELE.

Block (*Revue de Chirurgie,* Feb. 2, 1898) describes a new operation for the radical cure of hydrocele of the tunica vaginalis. The old method of injection of iodine, he points out, causes a very painful inflammatory reaction, and, in common with the more recent treatment by incision and drainage, necessitates prolonged rest in bed, and does not insure freedom from relapse. The author makes a free incision into the sac, applies a three-per-cent. solution of carbolic acid to the surface of the exposed testicle and the whole of the inner surface of the tunica vaginalis, and stuffs the cavity with strips of iodoform gauze. After removal of the gauze, on the third or fourth day, the wound in the skin is closed by catgut sutures. Of eighteen cases treated by this method, the patients having been seen after intervals between eight months and five years from the date of operation, in one only was a relapse noted. This was a case of very large hydrocele in a man aged sixty-four years.—*British Medical Journal, March 12, 1898.*

---

PLASTER of Paris sets much more efficiently with sulphate of potash than with chloride of sodium. The potash salt may be used in any strength; the stronger the solution the quicker the setting. —*Ex.*

